# Overlap of Adult-Onset Still Disease and Kikuchi-Fujimoto Disease: A Case Report and Literature Review

**DOI:** 10.7759/cureus.73283

**Published:** 2024-11-08

**Authors:** Juan Carlos Donaire-Castaños, Pablo Demelo-Rodríguez, Lucía Ordieres-Ortega, Susana Pardo-Sánchez, Francisco Galeano-Valle

**Affiliations:** 1 Department of Internal Medicine, Hospital General Universitario Gregorio Marañón, Madrid, ESP; 2 School of Medicine, Universidad Complutense de Madrid, Madrid, ESP

**Keywords:** histiocytic necrotizing lymphadenitis, kikuchi-fujimoto disease, overlap syndrome, still disease, yamaguchi criteria

## Abstract

Kikuchi-Fujimoto disease (KFD) and adult-onset Still disease (AOSD) are two rare conditions whose association poses a significant diagnostic challenge. KFD is characterized by subacute necrotizing lymphadenitis of unknown etiology, primarily affecting young adults, and often presents with fever and posterior cervical lymphadenopathy. AOSD is a systemic inflammatory disorder of unclear origin, defined by high-spiking fever, lymphadenopathy, hepatosplenomegaly, hyperferritinemia, and leukocytosis. To date, only 15 cases of the coexistence of both conditions have been reported, providing valuable insight into their pathophysiology.

We present the case of a 32-year-old Moroccan woman with prolonged fever, arthralgia, and axillary and mediastinal lymphadenopathy. After extensive evaluation, a lymph node biopsy confirmed the diagnosis of KFD, and she met the Yamaguchi criteria for AOSD. Treatment with oral prednisone was initiated, leading to rapid resolution of fever and normalization of acute-phase reactants. This case aligns with previously documented patterns in the literature, indicating a potential shared pathogenic mechanism. The lack of specific autoantibodies in these overlap cases emphasizes the need for clinicians to look for atypical clinical presentations when diagnosing these conditions. While corticosteroids have shown effective symptomatic control in the 16 reported cases, further research is necessary to explore targeted therapies, as many patients lack adequate follow-up regarding long-term management and response to treatment.

## Introduction

Kikuchi-Fujimoto disease (KFD), also known as histiocytic necrotizing lymphadenitis, is a rare condition characterized by subacute necrotizing lymphadenopathy, often associated with fever. Clinicians and pathologists are generally unfamiliar with this rare entity, which frequently presents significant diagnostic challenges [1-3]. Adult-onset Still disease (AOSD) is a systemic inflammatory disorder of unknown etiology, with a pathogenesis that is not yet fully understood; it is characterized by high-spiking fever, lymphadenopathy, hepatosplenomegaly, hyperferritinemia, and leukocytosis [4,5]. Fifteen prior cases of KFD and AOSD co-occurring have been reported in the medical literature [6-17]. These overlaps, along with certain distinguishing clinical and laboratory characteristics in these co-occurrences, offer insight into the pathophysiology of both rare disorders. Here, we present an additional case of KFD/AOSD overlap in a young Moroccan female patient, along with a literature review to further analyze and compare the reported cases to date.

## Case presentation

A 32-year-old woman from Morocco, residing in Spain since 1998, with no significant medical or surgical history, presented to the emergency department in August 2019 on four occasions over the previous month due to daily febrile peaks up to 40°C, accompanied by chills, hyporexia, a 7 kg weight loss, pharyngitis, and arthralgia in the proximal interphalangeal joints. She denied any relevant epidemiological history, including recent travel, contact with animals, or outdoor excursions. Additionally, none of her household members had experienced recent infectious episodes. Upon arrival, physical examination revealed a temperature of 38.5°C, blood pressure of 112/69 mmHg, heart rate of 120 bpm, oxygen saturation of 97%, and otherwise unremarkable findings. Laboratory tests showed hemoglobin of 11.1 g/dL (normal range: 12-16 g/dL), C-reactive protein (CRP) of 12.6 mg/dL (normal range: <0.4 mg/dL), fibrinogen of 519 mg/dL (normal range: 150-450 mg/dL), and an erythrocyte sedimentation rate (ESR) of 40 mm (normal range: 2-20 mm) in the first hour. No significant alterations were detected in the remaining parameters of the complete blood count, coagulation, and biochemistry. A chest X-ray was normal, and blood and urine cultures were sterile. The patient was admitted to the Internal Medicine ward due to a fever of unknown origin.

During hospitalization, she continued to experience daily febrile peaks despite antipyretic treatment and developed bilateral axillary lymphadenopathies by the seventh day of hospitalization. Protein electrophoresis and tumor markers (alpha-fetoprotein, carcinoembryonic antigen, CA-125 antigen, CA-15-3 antigen, CA-19-9 antigen, CYFRA 21-1, and beta-2-microglobulin) were normal. Autoantibodies, including antinuclear antibodies (ANA) and rheumatoid factor, were negative. Serologies for human immunodeficiency virus, Epstein-Barr virus, varicella-zoster virus, cytomegalovirus, hepatitis C, B, and A viruses, measles, mumps, rubella, syphilis, *Leishmania*, *Rickettsia*, *Legionella*, *Chlamydia*, *Mycoplasma*, *Toxoplasma*, and Rose Bengal agglutination were negative. Polymerase chain reaction (PCR) testing for *Tropheryma whipplei*, *Bartonella *spp., and *Coxiella burnetii* was normal.

An interferon-gamma release assay was positive. Although acid-fast bacilli (AFB) staining and PCR for *Mycobacterium tuberculosis* in urine, sputum, and blood were negative, antituberculosis treatment (isoniazid, rifampicin, ethambutol, and pyrazinamide) was initiated on the 10th day of hospitalization. Simultaneously, blood tests revealed a significant increase in lactate dehydrogenase (LDH) (1,015 U/L), ferritin (5,117 ng/mL), and transaminases (alanine aminotransferase (ALT): 1,000 U/mL). Given that the antituberculosis treatment could be contributing to the hepatic toxicity, it was discontinued. However, LDH, ferritin, and ALT levels did not decrease in the following weeks (Table 1).

**Table 1:** Laboratory results ESR: erythrocyte sedimentation rate, CRP: C-reactive protein, LDH: lactate dehydrogenase, ALT: alanine transaminase

Parameter	Result	Reference value	Units
Hemoglobin	11.1	11.6-15.0	g/dL
ESR	40	<19	mm/hour
Fibrinogen	519	200-400	mg/dL
CRP	12.6	<0.3	mg/dL
LDH	1,015	125-220	U/L
Ferritin	5,117	30-400	ng/mL
ALT	1,000	7-56	U/mL

An abdominal ultrasound and a transthoracic echocardiogram were normal. A thoracoabdominal computed tomography (CT) scan revealed multiple lymphadenopathies in the mediastinal and axillary chains, along with a small consolidation in the right lower lobe, suggesting tuberculosis as the primary possibility, although lymphoma could not be ruled out. A fiber-optic bronchoscopy was performed to obtain tissue samples via fine needle aspiration of lymphadenopathy, which was once again negative for tuberculosis (both AFB and PCR) and inconclusive to any other disease.

**Figure 1:**
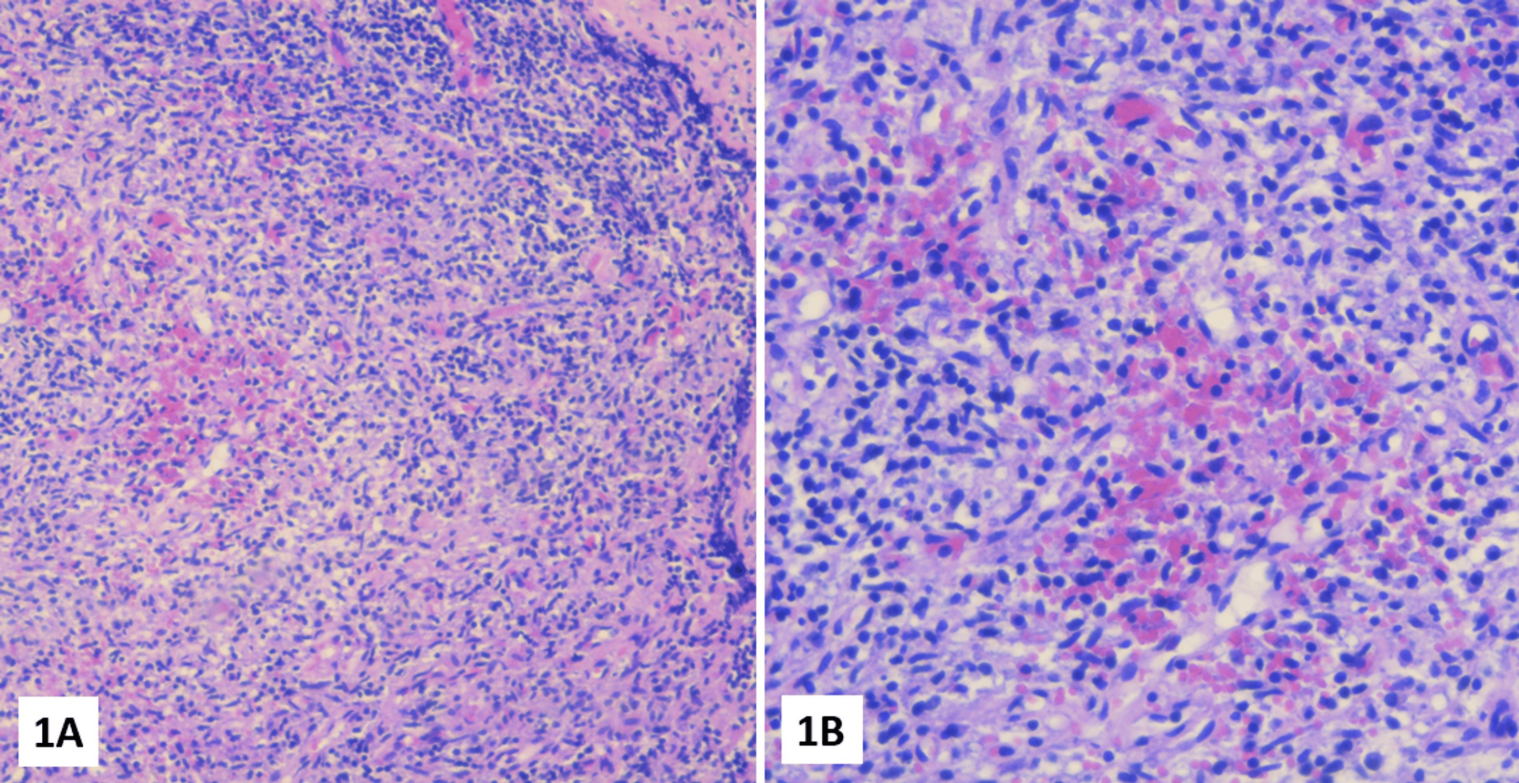
An excisional biopsy of an axillary lymph node demonstrated reactive lymphoid follicles with expanded paracortex and extensive paracortical karyorrhexis and necrosis 1A: Hematoxylin and eosin stain, original magnification: ×100. 1B: Hematoxylin and eosin stain, original magnification: ×1,000.

The lymphadenopathies rapidly increased in size, and daily fever persisted on the 24th day of hospitalization. Finally, a complete lymph node biopsy was performed two days later, revealing areas of necrosis and vascular proliferation consistent with KFD (Figure 1).

The patient also met Yamaguchi criteria [18] for AOSD (fever for >1 week, arthralgias for >2 weeks, pharyngitis, lymphadenopathies, abnormal liver function, and negative ANA). Consequently, treatment with oral prednisone 50 mg was initiated. The fever resolved within 48 hours, acute-phase reactants normalized, and liver function gradually returned to normal after one week. A positron-emission tomography-CT performed two weeks later showed resolution of the lymphadenopathies. The patient was discharged with a tapering dose of prednisone. After five years of follow-up, the patient remains asymptomatic.

## Discussion

Rai et al. performed a systematic literature review, identifying 10 previous cases and reporting an additional patient in May 2021 [16]. In addition to the cases described by Rai et al., we have found four more cases during our systematic review. Therefore, to the best of our knowledge, only 16 cases of the association between KFD and AOSD have been reported to date (Table 2).

**Table 2:** Reported cases of AOSD/KFD overlap AOSD: adult-onset Still disease, KFD: Kikuchi-Fujimoto disease, NSAIDs: nonsteroidal anti-inflammatory drugs, ESR: erythrocyte sedimentation rate, SLE: systemic lupus erythematosus, IL-6: interleukin-6

Nº	Author	Age	Gender	Symptoms	Laboratory/pathology	Treatment	Development
1	Ohta et al. (1988) [6]	41	Male	Fever, erythematous papules, arthritis	Leukocytes: 4,300/mm^3^, anemia, necrotizing lymphadenitis	NSAIDs, steroids	1 year; improvement with steroids
2	Ohta et al. (1988) [6]	14	Female	Spiking fever, rash, arthritis	Leukocytes: 19,900/mm^3^, hyperferritinemia, necrotizing lymphadenitis	Steroids	3 weeks; improved with steroids
3	Ohta et al. (1988) [6]	19	Male	Spiking fever, arthritis, xerostomia	Leukocytes: 9,800/mm^3^, ESR elevated, necrotizing lymphadenitis	NSAIDs, steroids	1 year; improved with steroids
4	Lyberatos (1990) [7]	18	Male	Fever, rash, arthritis	Leukocytes: 28,000/mm^3^, anemia, necrotizing lymphadenitis	Steroids	4 months; asymptomatic post-steroids
5	Lyberatos (1990) [7]	47	Female	Fever, rash, arthritis	Necrotizing lymphadenitis	Acetylsalicylic acid	4 years; mild symptoms
6	Garazzi et al. (1997) [8]	24	Female	Fever, urticarial lesions, arthritis	Leukocytes: 18,000/mm^3^, ESR elevated, necrotizing lymphadenitis	NSAIDs, methotrexate	10 years; improved with methotrexate
7	Cousin et al. (2001) [9]	32	Male	Fever, papules, arthritis	Leukocytes: 12,500/mm^3^, hyperferritinemia, necrotizing lymphadenitis	Acyclovir, steroids	8 months; improved with steroids
8	Ambrocio et al. (2006) [10]	57	Male	Fever, rash, arthritis	Leukocytes: 12,400/mm^3^, ESR elevated, necrotizing lymphadenitis	NSAIDs, steroids	4 months; improved with steroids
9	Kang et al. (2007) [11]	27	Female	High fever, rash, lymphadenopathy	Leukocytes: 12,210/mm^3^, hyperferritinemia, necrotizing lymphadenitis	NSAIDs, steroids	3 months; resolved with steroids
10	Miura et al. (2012) [12]	21	Female	Fever, rash, arthralgia	Leukocytes: 20,800/mm^3^, hyperferritinemia, necrotizing lymphadenitis	Steroids	8 months; improved with steroids
11	Toribio et al. (2015) [13]	22	Male	Fever, rash, arthritis	Leukocytes: 19,000/mm^3^, hyperferritinemia, SLE	NSAIDs, anakinra	8 months; resolved with anakinra
12	Sondermann et al. (2015) [14]	32	Female	Fever, rash, arthralgia	Leukocytes: 35,000/mm^3^, IL-6 elevated, necrotizing lymphadenitis	Antibiotics, steroids	4 weeks; resolved with steroids
13	Kodithuwakku et al. (2020) [15]	40	Male	Fever, cough, rash	Leukocytes: 34,400/mm^3^, hyperferritinemia, necrotizing lymphadenitis	NSAIDs, steroid pulses	45 days; resolved with steroids
14	Rai et al. (2021) [16]	51	Female	Daily fever, rash, myalgia	Leukocytes: 12,200/mm^3^, hyperferritinemia, necrotizing lymphadenitis	NSAIDs, methotrexate	12 weeks; good response to NSAIDs
15	Gómez-Vargas et al. (2022) [17]	51	Female	Fever, arthralgia	Leukocytes: 15,700/mm^3^, hyperferritinemia, necrotizing lymphadenitis	NSAIDs, steroids	2 months; improved with steroids
16	Present case	32	Female	Daily fever, weight loss	Hyperferritinemia, anemia, necrotizing lymphadenitis	Steroids	Resolved in 48 hours; improved with steroids

KFD typically affects young adults of Asian ancestry, although cases have been reported worldwide. Despite numerous studies, the cause of KFD remains uncertain [1]. Patients most commonly present with posterior cervical lymphadenopathy (90%), frequently with concomitant involvement of axillary and/or supraclavicular lymph nodes, along with fever. Generalized lymphadenopathy is rarely reported, and other infrequent symptoms include weight loss, vomiting, headache, arthralgia, night sweats, upper respiratory symptoms, and sore throat. Hepatomegaly and splenomegaly are rare (less than 5% of cases). Extranodal involvement is uncommon [1]. It is believed to have three evolving phases: proliferative, necrotizing, and xanthomatous. While the etiology remains unknown, viral and autoimmune mechanisms have been proposed. No specific laboratory tests contribute to the diagnosis, as laboratory findings are typically unremarkable [1,2]. Consequently, the final diagnosis is histologically based on a lymph node excisional biopsy, which reveals paracortical foci of coagulative necrosis containing karyorrhectic debris, surrounded by numerous CD68+/myeloperoxidase (MPO)+ histiocytes, plasmacytoid dendritic cells, and a minority of CD8+lymphocytes and immunoblasts [3]. The differential diagnosis of KFD includes infectious lymphadenitis, systemic lupus erythematosus, and lymphoma [1].

The prevalence of AOSD ranges from one to 10 cases per million in Europe and Japan, respectively [19,20]. AOSD is traditionally characterized by four cardinal symptoms (skin rash, fever > 39°C, leukocytes > 10,000/mm^3^ with neutrophils > 80%, and arthritis/arthralgia), although many other manifestations may also be present (odynophagia, myalgia/myositis, lymphadenopathy, splenomegaly, serositis, hepatitis, elevated ESR and CRP levels, elevated ferritin, decreased glycosylated ferritin, and coagulation disorders) [5]. The clinical course can be divided into three significant patterns, each with a different prognosis: self-limited or monophasic, intermittent or polycyclic systemic, and chronic articular. The diagnosis is based on a characteristic constellation of symptoms (high daily fever, arthralgia and arthritis, evanescent salmon-pink skin rash, sore throat, myalgias, lymphadenopathies, and splenomegaly) and the exclusion of infections, hematological malignancies, and alternative rheumatological conditions [21].

No single clinical sign or laboratory abnormality is sufficiently specific to confirm a diagnosis of AOSD. Therefore, classification criteria are often used, although they were primarily developed for research rather than for diagnostic purposes. Two sets of criteria have been validated. The Yamaguchi criteria are the most widely used [18], but they include exclusion criteria such as infections, malignancies, and other rheumatic diseases and should only be applied after a broad diagnostic workup, which can be challenging in clinical practice (Table 3). The Fautrel criteria, which include ferritin and glycosylated ferritin levels as diagnostic biomarkers and do not require exclusion criteria, offer an alternative (Table 4) [22]. In a 2018 validation study, both sets showed high sensitivity and specificity [21].

**Table 3:** Yamaguchi criteria for classification of adult-onset Still disease Source: [18]

Yamaguchi criteria		
Five or more criteria are required, of which two have to be major.		
Major criteria	Minor criteria	Exclusion criteria
Fever > 39°C, lasting 7 days or longer	Sore throat	Infections
Arthralgia or arthritis, lasting 14 days or longer	Recent development of significant lymphadenopathy	Malignancies (mainly malignant lymphoma)
Typical rash	Hepatomegaly or splenomegaly	Other rheumatic disease (mainly systemic vasculitides)
Leukocytosis > 10,000/mm^3^ with >80% polymorphonuclear cells	Abnormal liver function tests	-
-	Negative tests for antinuclear antibody (IF) and rheumatoid factor (IgM)	-

**Table 4:** Fautrel criteria for classification of adult-onset Still disease Source: [22]

Fautrel criteria	
Four or more major criteria are required, or 3 major and 2 minor criteria.	
Major criteria	Minor criteria
Spiking fever ≥ 39°C	Maculopapular rash
Arthralgia	Leukocytosis ≥ 10,000/mm^3^
Transient erythema	-
Pharyngitis	-
Polymorphonuclear cells ≥ 80%	-
Glycosylated ferritin ≤ 20%	-

Our case involved a young Moroccan woman with a four-week history of high fever, arthralgia, pharyngitis, mediastinal and axillary lymphadenopathy, elevated transaminases, and negative rheumatoid factor and antinuclear antibodies. She met two major and four minor Yamaguchi criteria with no exclusion criteria, yielding 96.3% sensitivity and 98.2% specificity for an AOSD diagnosis [18]. Additionally, using the modified Yamaguchi criteria (elevated ferritin), the sensitivity reaches 100% and specificity 97.1% [22]. According to the Fautrel criteria, the patient met three major and no minor criteria [22].

Therefore, our index case follows the clinical pattern observed in previously published cases. As developed by Rai et al., the diagnostic approach to overlap syndromes in systemic autoimmune diseases is based on the association of specific antibody markers and clinical patterns [16]. In the KFD/AOSD overlap, the absence of suggestive laboratory markers necessitates the search for unique or atypical clinical features in the individual diseases' typical presentation [23]. For instance, in the KFD case series by Nakamura et al., only 3% of patients had generalized lymphadenopathy [24], whereas in the overlap case series, this feature was present in eight of the described patients [16]. Similarly, joint involvement is rare in KFD but is more common in AOSD. In the overlap cases, joint involvement was symmetrically distributed in both sexes, typical of AOSD [13]. Pathological analysis of lymph node biopsies showed necrotizing lymphadenitis, characteristic of KFD, in 13 of the reported cases. However, in previous series, these findings are uncommon in AOSD, occurring in less than 8% of reported cases [25]. Thus, the clinic-pathological pattern is not characteristic of either KFD or AOSD individually, but their combined clinical presentation follows a distinct pattern in the 14 reported cases, fulfilling the diagnostic criteria for both diseases. Therefore, consistent with the literature [1,16], the KFD/AOSD overlap and their similar response to anti-inflammatory and immunosuppressive treatment suggest a shared aetiopathogenic mechanism. Despite limited studies on cytokines involved in overlap cases, multiple inflammatory markers have been described for both entities individually, such as interferon-gamma (IFN-γ) for AOSD and interleukin-1 beta (IL-1β) for KFD, raising hypotheses about potential therapeutic targets [13]. Nevertheless, there is insufficient evidence to recommend treatments targeting specific cellular pathways beyond corticosteroids, which have achieved clinical remission in all reported cases, including two in which immunosuppressants such as methotrexate were used [8,16].

## Conclusions

Still disease and Kikuchi-Fujimoto disease are two rare conditions, and their association presents a significant diagnostic challenge. Although this overlap is extremely rare and there is limited information about the best treatment and prognosis for such patients, our recommendations align with the reviewed literature. For diagnostic purposes, in the absence of specific autoimmunity markers, we recommend searching for atypical clinical patterns in each disease's classic presentation. Therefore, the diagnosis of KFD/AOSD overlap syndrome was based on a thorough medical history, the histopathological description of lymph nodes, and the response to corticosteroid treatment. Regarding treatment, despite the aetiopathogenic hypotheses, there is not enough evidence to recommend therapies targeting specific cellular mechanisms. In the 16 reported cases, including the index case, the response to corticosteroids or immunosuppressants was adequate for symptom control. However, many patients were not followed up, and the response to steroid-sparing agents was not described.
